# Weaving the health and pharmaceutical care agenda through the themes of the commonwealth heads of government meeting (CHOGM), London 2018

**DOI:** 10.1186/s40545-018-0140-3

**Published:** 2018-04-11

**Authors:** Victoria Rutter, Amy Hai Yan Chan, Chloe Tuck, Lina Bader, Zaheer-Ud-Din Babar, Ian Bates

**Affiliations:** 1 Commonwealth Pharmacists Association, 66-68 East Smithfield, Whitechapel, London, UK; 2 School of Pharmacy, University College London, London, UK; 3 Spoonful of Sugar Ltd, London, UK; 4 International Pharmaceutical Federation, The Hague, Netherlands; 5 University of Huddersfield, Queensgate, Huddersfield, UK

**Keywords:** Commonwealth, Health, Heads of government, Prosperity, Sustainability, Fairness, Security, Partnerships

## Abstract

The biennial Commonwealth Heads of Government Meeting (CHOGM) this year is based around four key themes: prosperity, fairness, sustainability and security. This is an opportune time to consider the role of pharmacists in healthcare delivery, and particularly their contribution to achieving the United Nations Sustainable Development Goals (SDGs). As a member of the Commonwealth Health Professions Alliance (CHPA), the Commonwealth Pharmacists Association (CPA) has been working to ensure that pharmacy-related aspects of health are represented in the advocacy papers submitted by Civil Society. Echoing the CHPA’s priorities around SDG 3 (health) and the attainment of sustainable universal health coverage (UHC), the CPA has been raising the profile of key priorities for our members, including: addressing the shortage of healthcare workers (with an emphasis on pharmacists); need for access to quality medicines and medicines information; tackling anti-microbial resistance and substandard/falsified medicines; and championing the role of digitalisation and partnerships. This editorial discusses how the work of the CPA links with the themes of CHOGM, the CPA’s input into this meeting and the direction of travel ‘Towards a Common Future’ for health and pharmacy in the Commonwealth.

The biennial Commonwealth Heads of Government Meeting (CHOGM) will be hosted in London on 16th–20th April 2018, with a theme of ‘Towards a Common Future’. The meeting will provide a platform to discuss current and common issues affecting the 53 Commonwealth nations, strengthening efforts to tackle these together [1]. The themes and priorities of the 2018 CHOGM are prosperity, fairness, sustainability and security [2]. These themes were announced last year by the UK’s Prime Minister Teresa May, and the Prime Minister of Malta Dr. Joseph Muscat, with the Commonwealth Secretary-General. Papers have been submitted to the meeting around these themes with an additional theme invited around “collaboration and partnership”, emphasizing the collaborative nature of the Commonwealth and the role of sustainable partnerships for delivering key priorities. As a member of the Commonwealth Health Professions Alliance (CHPA), the Commonwealth Pharmacists Association (CPA) has been working with other Civil Society organizations to ensure that health is represented in the submissions. This editorial summarizes how the CPA’s mission to support the better use of medicines aligns with the CHOGM key themes and health agenda.

## Prosperity

Effective provision of and access to appropriate standards of healthcare and education underpin prosperous societies. Although many definitions of prosperity exist, it is clear that prosperity cannot be achieved without first having a healthy and educated population [3, 4]. Achieving Universal Health Coverage (UHC) through effective use of medicines, and strengthened health systems is essential to protect the prosperity, health and security of people globally. This is something that needs to be continually highlighted as health and education are often not prioritized for investment, with resources being provided to areas that are more obviously related to prosperity, such as trade.

## Fairness

Reducing health inequalities by ensuring that all people have access to affordable and appropriate healthcare and medicines is a definitive part of the fairness agenda, and linked to better health outcomes [5].Achieving Universal Health Coverage (UHC) for all members is linked to the United Nation’s (UN) Sustainable Development Goals (SDGs), which is a collection of 17 global goals set by the UN [6]. UHC specifically relates to SDG 3: ‘*good health and wellbeing for all people*’, and has been the focus of many Commonwealth Health Ministers’ meetings. The UK has one of the most well established UHC systems in the world, but is not the only successful model. Sri Lanka’s health system provides an example of how UHC can be achieved with limited resources, resulting in impressive outcomes [7].For UHC to be achieved and sustainable, there are multiple aspects that need to be addressed and invested in.

## Sustainability

Access to adequate resources required for effective health systems is a key part of achieving sustainable progress towards UHC [8]. This includes appropriately trained healthcare professionals and resources that support the delivery of a quality service to the public, including quality medicines and medicines information. Alongside access to and sustainable financing of UHC, developing the health workforce will be one of the areas of focus for health at the 2018 People’s Forum that runs alongside the CHOGM.

### Developing the health workforce

By 2030, there will be an estimated 18 million shortfall in healthcare workers, disproportionately concentrated in low and middle-income countries (LMICs) [9]. Urgent action is required to transform our health workforce and fulfil the aspirations of SDG 3 [10]. Access to effective and safe medicines is a central pillar of any functioning healthcare system. Medicines remain the most frequent healthcare intervention worldwide, and national medication expenditure is second only to human resource costs in healthcare systems [11]. Expenditure is set to rise further as the burden of non-communicable diseases (NCDs) increases in the globally aging population. As medicines experts, the pharmaceutical workforce play a key role in improving health outcomes through optimizing use of medicines [12, 13]. Delivering effective medicines-related health services relies on investing in the development of an adaptable, competent and well-distributed pharmaceutical workforce that can contribute towards achieving UHC, SDGs, and strengthening health systems [14].

In November 2016, the International Pharmaceutical Federation (FIP) launched the Pharmacy Workforce Development Goals (PWDGs) alongside a global vision for the development of the pharmaceutical workforce [15]. The PWDGs are a set of measurable, feasible and tangible goals to implement transformative change for the global pharmaceutical workforce. The goals are directly aligned with major international healthcare policies and workforce strategies, and the CPA is working in collaboration with FIP’s education division to support national pharmacy associations throughout the Commonwealth towards national-level implementation of these goals.

One example of how the CPA is supporting national pharmacy associations to achieve the PWDGs and address the shortfall of appropriately skilled pharmacists [9] is by developing sustainable models of post-graduate, pan-Commonwealth training partnerships. The Tropical Health Education Trust’s (THET) has a successful Health Partnership Scheme (HPS), which could provide a model for pharmacist training partnerships. THET and the CPA share a joint vision to see more pharmacists included in HPS. WHO’s 3rd global patient safety challenge: tackling medication-related harm, [16] aims to reduce medication errors by 50% in 5 years - it is opportune timing to increase the pharmaceutical workforce to help achieve this goal.

### Ensuring access to reliable and independent resources of medicines information

Having access to reliable and independent medicines information is key for the provision of evidence-based care and achieving positive health outcomes, [17] yet many healthcare providers do not have access to quality medicines information. The CPA’s PharmAid scheme (the redistribution of recent editions of medicines information resources to LMICs in the Commonwealth) has sent over 200,000 books to 28 Commonwealth countries since 2005. In a recent survey, 80% of PharmAid recipients stated that the resources provided through PharmAid were very useful, or their practice would not be the same without them.

## Security

### Response to pandemics and infectious disease

The recent Ebola and Zika outbreaks are clear examples of how pandemics can present a threat to global health security. The WHO has declared that antimicrobial resistance (AMR) is also global health security threat, [18] and it is estimated that people are more likely to die from drug-resistant infections than cancer by 2050 if AMR is not addressed immediately. This issue is magnified in LMICs, where the incidence of AMR is much higher and significantly under-reported. In September 2016, 193 countries of the United Nations agreed to a landmark declaration to ‘rid the world of superbugs’, which all Commonwealth countries signed. Of these, 44% have a current action plan, 27% have an action plan under development, and 29% do not yet have an established plan [19].

The CPA has been raising awareness of AMR through their global campaigns, webinars, and resources to help National Pharmacy Associations and their members develop their own campaigns, adhere to best practices to reduce inappropriate use of antimicrobials [20]. The CPA have involved health ministers and their representatives through the Commonwealth Advisory Committee on Health (CACH) in Colombo 2016, and hosted presentations, workshops and discussions at international conferences in Australia and Sri Lanka, to ensure AMR is high priority.

A recent partnership between the CPA and health behaviour change specialists Spoonful of Sugar has been pivotal for understanding the behavioural drivers of AMR across the Commonwealth. Understanding and tackling behaviour is important in the battle against AMR. In LMICs, pharmacists may have motivations and underlying beliefs associated with running a business and maintaining customer loyalty [21, 22], particularly when patients seek antimicrobials [23, 24], that are discordant with antimicrobial stewardship (AMS) and drive inappropriate antimicrobial use.

### Substandard and falsified medicines

It is estimated that up to 50% of medicines in some parts of the world are substandard or falsified, with an estimated 700,000 people dying each year due to fake malaria and tuberculosis medication [25]. Regulation, monitoring and education are amongst the aspects that need to be addressed to tackle this. As partners of the Fight the Fakes – a campaign that aims to raise awareness on the dangers of fake medicines [26], the CPA has been advocating to highlight this issue in the Commonwealth through campaigns and advocacy efforts.

### The role of digitilisation

There is a need to support all member nations to ensure that there is a Commonwealth-wide surveillance capacity and networks to help detect, isolate, monitor and co-ordinate responses to potential disease outbreaks. It is hoped that the Commonwealth Digital Health Initiative (CWDIH), which was recently established by the Commonwealth Medical Association (CMA) and is due for launch during CHOGM 2018, will take on monitoring as one of its key roles. In addition to providing digital response around pandemics and AMR, the CWDIH could also improve reporting of substandard and falsified medicines. The wider implications of digitalisation on the delivery of medicines-related healthcare services, and the training of healthcare professionals, also need consideration.

## Partnerships

Partnership relates to SDG 17. The Commonwealth provides a network that could potentially support all countries to achieve UHC through collaborative working. The 2016 Colombo Declaration highlighted the commitment of organizations to work together to deliver an action plan to improve the health and wellbeing of the Commonwealth. Similarly, delivery of agreed actions from CHOGM could be supported through pan-Commonwealth partnerships. Combining expertise to create a multi-disciplinary, pan-Commonwealth, public-private sector approach will be crucial to tackle the issues facing health and wellbeing today and in the future.

## Summary

The upcoming CHOGM is a key opportunity to reenergize the Commonwealth and empower the people towards a shared goal of building a common future to improve health. In terms of the joint aspirations of achieving UHC, SDGs and strengthening health systems, investment in the pharmacy workforce using a pan-Commonwealth partnership approach in collaboration with pharmacist professional bodies such as the CPA is a positive and welcome step in the right direction.
